# Which, how, and what? Using digital tools to train surgical skills; a systematic review and meta-analysis

**DOI:** 10.1016/j.sopen.2023.10.002

**Published:** 2023-10-04

**Authors:** Tim M. Feenstra, Sebastiaan L. van der Storm, Esther Z. Barsom, Jaap H. Bonjer, Els J.M. Nieveen van Dijkum, Marlies P. Schijven

**Affiliations:** a Department of Surgery, Amsterdam UMC location University of Amsterdam, Amsterdam, the Netherlands; b Amsterdam Gastroenterology, Endocrinology and Metabolism, Amsterdam, the Netherlands; c Amsterdam Public Health, Digital Health, Amsterdam, the Netherlands; d Cancer Centre Amsterdam, Amsterdam UMC, University of Amsterdam, Amsterdam, the Netherlands; e Amsterdam institute for Infection and Immunity, Amsterdam, the Netherlands

**Keywords:** Residency, Skills, Training, Digital training, Systematic review, Meta-analysis

## Abstract

**Background:**

Digital tools like digital box trainers and VR seem promising in delivering safe and tailored practice opportunities outside of the surgical clinic, yet understanding their efficacy and limitations is essential. This study investigated *Which* digital tools are available to train surgical skills, *How* these tools are used, *How* effective they are, and *What* skills they are intended to teach.

**Methods:**

Medline, Embase, and Cochrane libraries were systematically reviewed for randomized trials, evaluating digital skill-training tools based on objective outcomes (skills scores and completion time) in surgical residents. Digital tools effectiveness were compared against controls, wet/dry lab training, and other digital tools. Tool and training factors subgroups were analysed, and studies were assessed on their primary outcomes: technical and/or non-technical.

**Results:**

The 33 included studies involved 927 residents and six digital tools; digital box trainers, (immersive) virtual reality (VR) trainers, robot surgery trainers, coaching and feedback, and serious games. Digital tools outperformed controls in skill scores (SMD 1.66 [1.06, 2.25], *P* < 0.00001, I^2^ = 83 %) and completion time (SMD -1.05 [−1.72, −0.38], *P* = 0.0001, I^2^ = 71 %). There were no significant differences between digital tools and lab training, between tools, or in other subgroups. Only two studies focussed on non-technical skills.

**Conclusion:**

While the efficacy of digital tools in enhancing technical surgical skills is evident - especially for VR-trainers -, there is a lack of evidence regarding non-technical skills, and need to improve methodological robustness of research on new (digital) tools before they are implemented in curricula.

**Key message:**

This study provides critical insight into the increasing presence of digital tools in surgical training, demonstrating their usefulness while identifying current challenges, especially regarding methodological robustness and inattention to non-technical skills.

## Highlights

• There are six digital tools available to train surgical skills
• Digital tools’ effectivity is comparable to training in a wet/dry lab and more effective compared to a control group
• Most evidence is available for VR trainers which are equally effective as box trainers and training in a wet/dry lab
• The methodological robustness of studies needs to improve, before digital tools are utilized based on educational research
• Unlike technical skills, evidence on training non-technical skills digitally is lacking, revealing an educational blind spot.

## Introduction

Surgical residents need sufficient clinical training experiences to develop their skills, achieve proficiency, and ultimately become competent surgeons. While clinical training is critical to achieve these goals, it is affected by available case-load, exposure, and most importantly, patient safety [1,2]. As a result, residents also need training outside of the daily clinical practice and operating rooms (OR) which can be tailored to their educational needs, and provide them with the opportunity to practice and learn from mistakes without endangering patients [3,4].

Digital tools, such as virtual reality (VR), digital box trainers, and applications for mobile platforms (apps), can provide these training opportunities, and are increasingly used by surgical educators – especially since the COVID-19 pandemic [[5], [6], [7], [8], [9]]. There are myriad studies that introduce or validate a digital tool, and several reviews which evaluate these tools based on the technology used [[10], [11], [12], [13], [14]]. However, before these tools are implemented in surgical curricula and relied on to improve training, an overview of available tools, their merits, and the skills they aim to train is essential – and currently missing.

Technical skills are an important aspect of surgical training, well incorporated in surgical curricula, and widely discussed in literature. Conversely, although non-technical skills have been shown to negatively affect performance and surgical outcome, they are often regarded as being more difficult to identify and teach [[15], [16], [17], [18], [19]]. Therefore, this systematic review and meta-analysis aims to answer the following three questions: *Which* digital tools are available to train surgical skills and what is their efficacy, *How* are these tools used, and *What* skills (technical and/or non-technical) do these tools aim to train?

## Material and methods

This systematic review and meta-analysis was performed in accordance with the *Cochrane Handbook for Systematic Reviews of Interventions* version 6.0 and PRISMA-guidelines [20,21].

### Literature search

MEDLINE, EMBASE, and Cochrane databases were reviewed for studies assessing digital skill training tools for surgical residents, published since January 1st 2010 up until the last search update of December 7th, 2022. Keywords related to digital training, skills, and competencies were incorporated in the search, the full search can be found in the **Supplementary Material**. Included articles were cross-referenced for additional relevant studies. Digital training was defined according to the European Commission definition: the pedagogical use of digital technologies to support and enhance learning, teaching and assessment [22]. Skills were defined according to Merriam-Webster dictionary: “a learned power of doing something competently: a developed aptitude or ability” [23].

Randomized clinical trials (RCTs) were included in this review to attain the highest level of evidence and to enable comparison of digital tools. RCTs were eligible if they were published in Dutch or English, assessed digital training tools aimed at skill acquisition, and used objective performance indicators such as computed metrics or scoring tools. Studies which used subjective outcomes, such as participant questionnaires or self-evaluation tools, were excluded. Additionally, studies reporting on conference proceedings, study protocols, and studies which evaluated multiple digital tools without assessing each source separately were excluded. Two authors (TM and SvdS) assessed all titles and abstracts and included studies for full-text appraisal when both reviewers agreed on inclusion. Disagreements were resolved by consulting a third reviewer (MPS). A standardized form was used to systematically extract data from the studies including; trained skills, study design, characteristics of participants and digital tools, addressed skills, outcomes, and factors affecting the efficacy of the training tool.

### Data analysis and synthesis

#### Tool availability, efficacy, and use

Studies were categorized according to the digital tool they examined. Overall efficacy was evaluated through meta-analyses of post-test outcomes on skill scores (checklist scores and computed metrics) and time (task completion time). Based on these data, digital tools were compared with a control group (receiving traditional and/or no additional training), and with training in a wet or dry lab. Within these comparisons, subgroups were created based on the studied digital tool to evaluate the efficacy of individual tools and the heterogeneity therein. If sufficient studies were available, digital tools were compared to other digital tools. To examine how the utilization factors of digital tools affected outcomes, study data were pooled according to their training structure (self-directed versus prescribed training or training to proficiency) and training duration (minutes-days versus weeks-months).

Meta-analyses on pooled data were performed using Cochrane's Review Manager (RevMan) 5.4 [24]. All extracted data were converted to standardized mean differences (Hedges g effect size). When mean and standard deviation(SD) were not available, reported outcomes (*p*-values, median, range, *P*-value, and 95 % Confidence Interval (CI)) were used to estimate the effect size. If none of these data were provided, a study was excluded from the meta-analysis. A random-effects model was used in al analyses due to expected methodological (arising from the broad literature search) and statistical heterogeneity, which was quantified by calculating the I^2^ statistic. Effect sizes were presented with 95 % CI's and deemed significant if *P* < 0.05. Because this review presents the minimally available evidence, outcomes of meta-analyses were reported even in the light of high heterogeneity [21].

#### Skills trained using digital tools

Studies were evaluated based on the skills they primarily aim to train: technical skills, general non-technical competencies (according to the CanMEDS framework), and non-technical surgical skills (according to the NOTSS taxonomy) [25,26]. The CanMEDS framework identifies seven competencies (roles) each physician should master, based on the needs of the people they serve. The Medical Expert is identified as the role in which the six intrinsic roles are integrated: the Communicator, Collaborator, Leader, Health Advocate, Scholar, and Professional roles. The framework provides key- and enabling competencies, which were used to assess reported outcome measures in this review. The NOTSS taxonomy is aimed specifically at non-technical skills in the OR. The taxonomy defines four skill categories (situation awareness, decision making, communication & teamwork, and leadership), which are all subdivided in three elements. The NOTSS system handbook described these categories and elements in-depth, and was used to assess the primary outcome measures in this review [27]. A graphical overview of the CanMEDS framework roles and NOTSS taxonomy categories can be found in Table 1. TMF and SvdS evaluated which skills were trained in the study, and whether this skill was included as the primary outcome of the study or assessed in any way by the authors.

**Table 1:** Definition of the seven CanMEDS roles and four NOTSS competencies.

CanMEDS	Medical expert	Integrating all of the CanMEDS Roles in the provision of high-quality and safe patient-centred care
	Communicator	Forming relationships with patients and their families that facilitate the gathering and sharing of essential information for effective health care
	Collaborator	Working effectively with other health care professionals to provide safe, high-quality, patient-centred care
	Leader	Engaging with others to contribute to a vision of a high-quality health care system and take responsibility for the delivery of excellent patient care
	Health Advocate	Working with those they serve to determine and understand needs, speak on behalf of others when required, and support the mobilization of resources to effect change
	Scholar	Demonstrating a lifelong commitment to excellence in practice through continuous learning and by teaching others, evaluating evidence, and contributing to scholarship
	Professional	Commitment to the health and well-being of individual patients and society through ethical practice, high personal standards of behaviour, accountability to the profession and society, physician-led regulation, and maintenance of personal health
NOTTS	Situation awareness	Developing and maintaining a dynamic awareness of the situation in OR. Elements are gathering information, understanding information, and projecting and anticipating future state
	Decision making	Diagnosing the situation and reaching a judgement in order to choose an appropriate course of action. Elements are: considering options, selecting and communicating options, implementing and reviewing decisions
	Communication and teamwork	Working to ensure that the team has an acceptable shared picture of the situation and can complete tasks effectively. Elements are: exchanging information, establishing a shared understanding, coordinating team activities
	Leadership	Providing directions to the team, demonstrating high standards of clinical practice and care, and being considerate about the needs of individual team members. Elements are: setting and maintaining standards, supporting others, and coping with pressure

#### Methodological quality and bias

The methodological quality of the included studies was assessed using the revised Cochrane risk of bias tool for randomized trials (RoB 2 tool), which determines an overall risk of bias of randomized trials based on five bias domains; selection of reported result, measurement of outcome, missing outcome data, deviations from intended interventions, and randomization process [28].

## Results

**Fig. 1:**
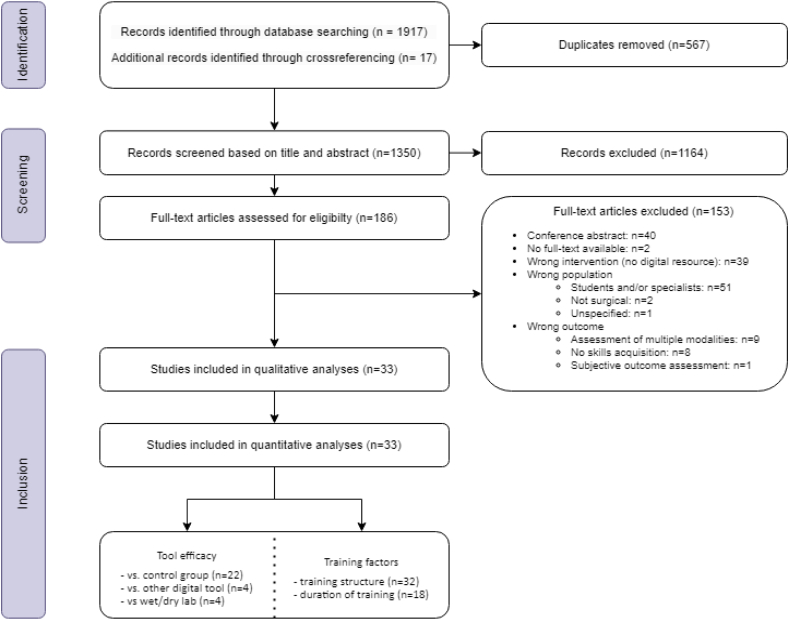
PRISMA flow diagram of included studies.

Eighteen hundred and fifty-one studies were screened based on title and abstract. A total of 178 full-texts were reviewed, resulting in the inclusion of 33 studies comprising 927 residents [[29], [30], [31], [32], [33], [34], [35], [36], [37], [38], [39], [40], [41], [42], [43], [44], [45], [46], [47], [48], [49], [50], [51], [52], [53], [54], [55], [56], [57], [58], [59], [60], [61]]. .Fig. 1 depicts the PRISMA flow diagram of included studies and Table 2 summarizes the study characteristics and describes demographics, study setting, and intervention protocols.

**Table 2:** Characteristics of included studies.

Author	Year	Country	Participants				Tools			Assessed skills		
			No	% female	Specialty	Level / experience (%)	Intervention(s)	Control	Other	Technical	CanMEDS^a^	NOTSS^a^
Ahlborg [29]	2013	Sweden	19	NS	Obstetrics and Gynaecology	Not specified	VR trainer	No additional training		Tubal occlusions	–	–
Akdemir [30]	2014	Turkey	60	25 %	Gynaecology	PGY 1 and 2	VR trainer	No additional training	Analogue box-trainer	Bilateral Tubal Ligation	–	(Decision making)
Araujo [31]	2014	Brazil	14	14,3 %	Surgery	No experience with laparoscopic colectomy	VR trainer	No additional training		Laparoscopic skills	–	(Decision making)
Borahay [32]	2013	USA	16	83,3 %	Obstetrics and Gynaecology	8 PGY 1 8 PGY 2	Robot trainer		Analogue box-trainer	Laparoscopic skills	–	–
Brown [33]	2017	USA	26	NS	10 General surgery, 7 Urology, 9 Obstetrics and Gynaecology	8 PGY 1 (30,1 %) 5 PGY 2 (19,2 %) 6 PGY 3 (23,1 %) 5 PGY 4 (19,2 %) 2 PGY 5 (7,7 %)	2 robot trainers			Basic robotic surgery skills	–	–
Camp [34]	2016	USA	57	NS	Orthopaedic surgery	9 PGY 1 (15,8 %) 12 PGY 2 (21,1 %) 6 PGY 3 (10,6 %) 9 PGY 4 (15,8 %) 9 PGY 5 (15,8 %)	VR trainer	No additional training	Cadaver training	Arthroscopy	–	(Decision making)
Cannon [35]	2014	USA	48	NS	Orthopaedic surgery	PGY 3	VR trainer	No additional training		Arthroscopy	–	–
Daly [36]	2013	USA	21	NS	Ophthalmology	PGY 2	VR trainer		Wet lab training	Cataract surgery	–	–
Dickerson [37]	2019	USA	42	26,2 %	Orthopaedic surgery	PGY 1–5 (mean PGY 2.3–2.7)	Post-op coaching session with POV-video of surgery	Post-op coaching session without POV-video of surgery		Intra-articular distal tibial fracture reduction	(Collaborator)	(Decision making)
Fried [38]	2010	USA	25	NS	Ear Nose Throat surgery	PGY 1–2	VR trainer	“Access to conventional material”		Endoscopic Sinus Surgery	(Communication and Teamwork)	
Garfjeld Roberts [39]	2019	UK	28	46,7 %	Trauma / orthopaedic surgery	24 PGY 2 (80 %) 6 PGY 3 (20 %)	Box trainer	“Normal deanery-provided training”		Knee arthroscopy	(Collaborator)	(Decision making)
Graafland [40]	2017	Netherlands	31	41.7 %	General surgery	3 PGY 1 (9,7 %) 20 PGY 2 (64,5 %) 1 PGY 3 (3,2 %)	Basic laparoscopic training course with serious game	Basic laparoscopic training course without serious game		Situational awareness in OR	(Communication and Teamwork)	
Hauschild [41]	2021	USA	38	NS	Orthopaedic surgery	PGY 1–5	VR trainer		Dry lab training	Shoulder arthroscopy	–	–
Hooper [42]	2019	USA	14	35.7 %	Orthopaedic surgery	PGY 1	Immersive VR trainer	“Standard study materials”		Shoulder Arthroscopy	–	Situation awareness
Hou [43]	2018	China	10	40 %	NS	No experience	VR trainer	“Traditional teaching method”		Cervical pedicle screw placement	–	(Decision making)
Huri [44]	2020	Turkey	34	0 %	Orthopaedic surgery	NS	VR trainer		Cadaver training	Shoulder Arthroscopy	(Collaborator)	(Decision making)
Jensen [45]	2014	Denmark	30	64.3 %	Urology, General surgery, Cardiothoracic surgery, Orthopaedic surgery	NS	VR trainer		Analogue box trainer	Shoulder Arthroscopy	(Communication and Teamwork)	
Kantar [46]	2020	USA	13	23.1 %	Plastic Surgery	3 PGY 1 (23,1 %) 3 PGY2 (23,1 %) 3 PGY 3 (23,1 %) 4 PGY 4 (30,8 %)	VR trainer		Learning from text-book	Unilateral cleft lip repair	–	–
Korets [47]	2011	USA	16	NS	Urology	10 PGY 1–3 (62,5 %) 6 PGY 4–5 (37,5 %)	Robot trainer with digital coaching vs. robot trainer with mentor	No additional training		Basic surgical skills	–	–
Korndorffer [48]	2012	USA	20	50 % (80 % digital, 20 % analogue)	General Surgery	PGY 1–5 (mean PGY 2.3–2.8)	Digital boxtrainer		Analogue boxtrainer	Basic laparoscopic skills	–	–
Kun [49]	2019	China	50	54 %	NS	PGY 2–3	Robot trainer with self-coaching with exercise videos	Robot trainer with self-coaching without videos of the exercise		Basic Robotic skills	(Collaborator)	(Decision making)
Logishetty [50]	2019	UK	28	29,2 %	Surgery Orthopaedic surgery	PGY 3–5	Immersive VR trainer	“Conventional preparatory materials”		Total hip arthroplasty	(Communication and Teamwork)	
Lohre [51]	2020	Canada	16	NS	Orthopaedic surgery	6 PGY 4 (37,5 %) 10 PGY 5 (62,5)	Immersive VR trainer	“Traditional learning using a technical journal article”		Shoulder arthroplasty	(Collaborator)	(Decision making)
McKinney [61]	2022	USA	22	NS	Orthopaedic surgery	7 PGY 1 (31,8 %) 7 PGY 2 (31,8 %) 3 PGY 3 (13,6 %) 3 PGY 4 (13,6 %) 2 PGY 5 (9,1 %)	Immersive VR trainer	“Reading the technique guide”		Knee arthroplasty	–	(Decision making)
Orzech [52]	2012	Canada	24	NS	General Surgery	PGY >2, median PGY 2.6–3.2	VR trainer		Analogue boxtrainer	Advanced laparoscopic skills	(Communication and Teamwork)	
Palter [53]	2013	Canada	16	NS	General Surgery	14 PGY 1 (87,5 %) 2 PGY 2 (12,5 %)	VR trainer	Normal residency curriculum, without additional training.		Basic laparoscopic skills Laparoscopic cholecystectomy	–	–
Sharifzadeh [60]	2021	Iran	46	100 %	Obstetrics and Gynaecology	PGY 2–3	Serious game	No additional training		Basic ynaecological skills	–	(Decision making)
Sloth [54]	2021	Denmark	46	69,6 %	General surgery Urology Gynaecology	PGY 1, no previous simulation training, <50 supervised laparoscopic procedures	Digital boxtrainer at home vs digital boxtrainer in hospital			Intracorporeal suturing	–	–
Valdis [55]	2016	Canada	40	30 %	General Surgery	< 10 h on robotic surgical simulator, mean year of training: 4–5	VR trainer	No additional training	Wet lab training Dry lab training	Basic robotic skills	(Decision Making)	
van Det [56]	2011	Netherlands	10	NS	General Surgery	No experience with laparoscopic surgery	Video-enhanced intraoperative feedback	Traditional intraoperative feedback		Nissen fundoplication	(Communication and Teamwork)	
Varras [57]	2020	Greece	20	45 %	Obstetrics and Gynaecology	< 10 laparoscopic surgeries, no experience with VR simulators	Digital boxtrainer VR trainer			Basic laparoscopic skills	–	Decision making
Waterman [58]	2016	USA	22	4,5 %	Orthopaedic surgery	PGY 1–4, median PGY 3.0	VR trainer	“Standard practice”		Basic laparoscopic skills	(Collaborator)	(Decision making)
Yiasemidou [59]	2017	UK	25	NS	General Surgery	ST3-ST4, <15 laparoscopic cholecystectomies as primary surgeon	VR trainer		Analogue box trainer	Laparoscopic cholecystectomy	(Communication and Teamwork)	

a When non-technical skills are presented between brackets, they were assessed by the authors but outcomes specific for that non-technical skill are not presented in the manuscript.

### Study characteristics and available tools

The 33 included studies addressed six digital tools;

1. Digital box trainers (*n* = 4, 12.1 %): Training box with a camera, instruments and training exercises, enhanced by digital computations of performance metrics.
2. Virtual Reality (VR) trainers (*n* = 18, 54.5 %): Computer- and screen-based software and hardware, which mimics surgical environments.
3. Immersive VR trainers (*n* = 4, 12.1 %): Computer-based system, which combines a VR-headset and handheld consoles to interact with digital surroundings.
4. Robot surgery trainers (n = 4, 12.1 %): Computer- and screen-based software and hardware, training robotic skills completely digital or in combination with analogue exercises.
5. Coaching and feedback (*n* = 5, 15.1 %): Tool which provides feedback on performed exercises, either by enabling recording and (re-)viewing of the exercise, or by analysing computed exercise metrics.
6. Serious games (*n* = 2, 6.1 %): “An interactive computer application … that has a challenging goal, is fun to play and engaging, incorporates some kind of scoring mechanism, and supplies the user with skills, knowledge or attitudes useful in reality” [62].

### Digital tools versus a control group

**Fig. 2:**
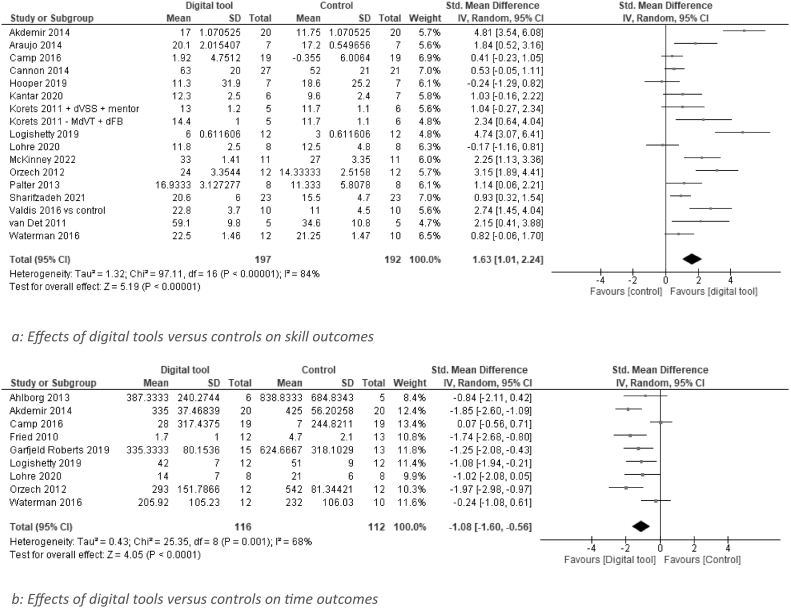
a: Effects of digital tools versus controls on skill outcomes b: Effects of digital tools versus controls on time outcomes.

**Fig. 3:**
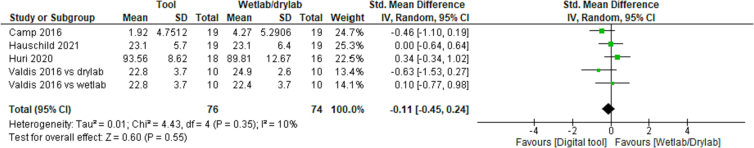
Effects of digital tools versus wet and dry lab on skill scores.

Twenty-three (70 %) studies compared digital tools with a control group which received traditional and/or no additional training [[29], [30], [31],^34^,^35^,[37], [38], [39], [40],^42^,^43^,^46^,^47^,[50], [51], [52], [53],^55^,^56^,^58^,^60^,^61^]. Seventeen of these were included in the meta-analysis based on skills [30,31,34,35,42,46,[50], [51], [52], [53],^58^,^61^] and nine were included in the meta-analysis based on time [29,30,34,38,39,[50], [51], [52],^58^]. In these analyses (Fig. 2, Fig. 3), residents using digital tools achieved higher skill scores (SMD 1.66[1.06, 2.25], *P* < 0.00001, I^2^ = 83 %) and required less time (SMD -1.05 [−1.72, −0.38], *P* = 0.0001, I^2^ = 71 %) than residents in a control group – although individual effect sizes varied widely and heterogeneity for both outcomes was high.

### Digital tools compared to wet lab and dry lab training

Of all studies, four (12.5 %) studies compared a digital tool with training in a wet and/or dry lab; three compared a VR trainer with wet/dry lab training [34,41,44]. Valdis et al. compared a robot trainer with training in both a wet lab and a dry lab [55]. .As depicted in Fig. 3, digital tools were equally effective with regard to skill scores (SMD -0.11 [−0.45, 0.24], *P* = 0.55, I^2^ = 10 %). Insufficient data was available to perform a comparison on skill completion time.

### Comparison of different tools: VR-trainer versus box trainers

**Fig. 4:**
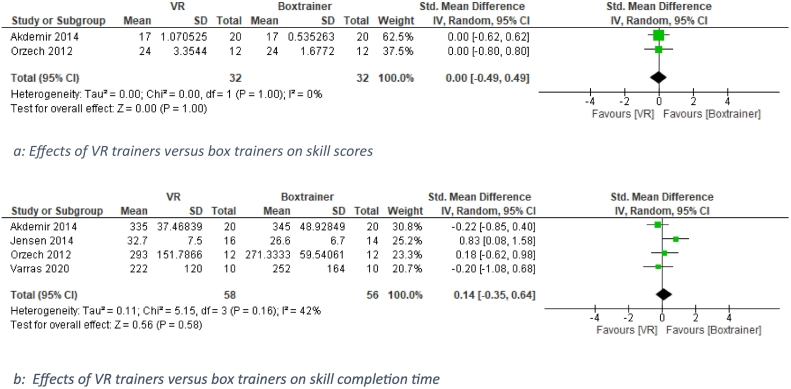
a: Effects of VR trainers versus box trainers on skill scores. b: Effects of VR trainers versus box trainers on skill completion time.

Four of the five (15.2 %) studies which compared a VR trainer with a box trainer were included in the analysis [30,45,48,52,57,59]. As depicted in Fig. 4, there were no significant differences between VR and box trainers in skills score (*n* = 2, SMD 0.00 [−0.49, 0.49], *P* = 1.00, I2 = 0 %) and skill completion time (*N* = 4, SMD 0.14 [−0.35, 0.64]. *P* = 0.58, I^2^ = 42 %).

### Subgroup analyses

Results of subgroup analyses are presented in Table 3, individual Forest-plots can be found in supplemental Figs. 2–7.

**Table 3:** Meta-analysis of subgroup analyses on skill scores and performance time.

	Number of studies	SMD [95 % CI]	P-value	I^2^-value
Tool subgroups				
Vs. control group - skill score				
Overall test for differences	16		0.32	15.1 %
VR trainer	8	1.63 [0.72, 2.54]	0.0004	87 %
Immersive VR trainer	4	1.56 [−0.42, 3.54]	0.12	91 %
Robot trainer	2	1.89 [0.22, 3.56]	0.03	70 %
Coaching and Feedback tool	2	2.24 [1.03, 3.46]	0.0003	0 %
Serious game	1	–	–	–
Vs control group – performance time				
Overall test for differences	9		0.93	0 %
Digital box trainer	1	–	–	–
VR trainer	6	-1.07 [−1.87, −0.28]	0.008	80 %
Immersive VR trainer	2	−1.05 [−1.72, −0.38]	0.0.002	0 %
Trainings factors subgroups				
Training structure				
Skill score (vs control group)	17		0.06	70.7 %
Prescribed	10	2.03 [1.02, 3.04]	<0.00001	88 %
Self-directed	7	1.06 [0.45,1.68]	0.0007	63 %
Performance time (vs control group)	9		0.36	0 %
Prescribed	6	−1.21 [−1.88, −0.54]	0.0004	62 %
Self-directed	3	−0.68 [−1.59, 0.22]	0.14	72 %
Training duration				
Skill score (vs control group)	16		0.06	70.7 %
Hours-days	9	1.11 [0.47, 1.75]	0.0006	74 %
Weeks-months	7	2.38 [1.19, 3.58]	<0.0001	88 %
Performance time (vs control group)	6		0.10	64.1 %
Hours-days	2	−0.21 [−1.05, 0.62]	0.61	38 %
Weeks-months	4	−1.12 [−1.79, −0.46]	0.0009	62 %

### Tool subgroups

Heterogeneity in outcomes of digital tools versus a control group is not explained by the different tools. There were no significant differences between subgroups in skill scores (*P* = 0.32, I^2^ = 15.1 %) and task completion time (*P* = 0.93, I^2^ = 0 %). Significant pooled effects of the tools on skill scores were observed for VR trainers (Skills: SMD 1.63 [0.72, 2.54], *P* < 0.00001, I^2^ = 87 %, time: SMD −1.07 [−1.87, −0.28], *P* = 0.0008, I^2^ = 80 %), robot trainers (skill: SMD 1.89 [0.22, 3.56], *P* = 0.03, I^2^ = 70 %), and coaching and feedback tools (skill: SMD 2.24 [1.03, 3.46], *P* = 0.0003, I^2^ = 0 %) – yet heterogeneity was a high for most of these outcomes. While pooled effects of using an immersive VR trainers were highly heterogeneous and not significant with regard to skills (SMD 1.56 [−0.42, 3.54], *P* = 0.12, I^2^ = 91 %), pooled effects on time were significant in the two studies assessing these outcomes (SMD 1.63 [0.72, 2.54], *P* < 0.00001, I^2^ = 87 %). Insufficient data was available for digital skills trainers and serious games.

### Training factors subgroups

Differences in training structure and training duration do not explain the heterogeneity in outcomes of digital tools versus a control group. Studies using a prescribed training structure (i.e. training for a defined amount of time or training to proficiency), achieved slightly higher final scores an needed slightly less time – but differences with a studies using a self-directed approach to using the digital tool were not significant (skill subgroup differences: *P* = 0.11, I2 = 61 %), time subgroup differences: *P* = 0.36, I2 = 0 %). The same differences were observed for pooled results based on training duration (hours to days versus weeks to months); while there were small differences between subgroup outcomes these differences were not significant (skill subgroup differences: *P* = 0.06, I2 = 70.7 %), time subgroup differences: *P* = 0.10, I2 = 64.1 %).

### Assessed skills

**Fig. 5:**
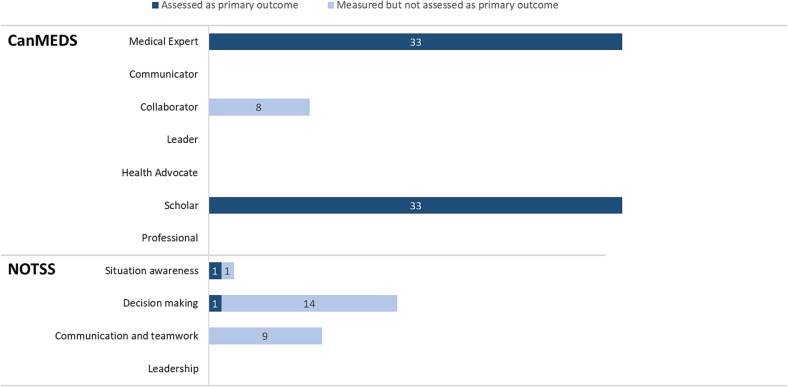
CanMEDS roles and NOTSS components in included studies.

Only Graafland et al. and Lohre et al. used non-technical skills in their primary outcomes; situation awareness and decision making, both within the NOTSS framework (Fig. 5) [40,51]. Components of the ‘Medical Expert’ and ‘Scholar’ CanMEDS roles overlapped with technical skills trained and measured by all other studies. Fifteen (54.5 %) studies used skills checklists, such as the OSATS (Objective Structured Assessment of Technical Skills), ASSET (Arthroscopic Surgical Skill Evaluation Tool), and GOALS (Global Operative Assessment of Laparoscopic Skills). These checklists include non-technical skills such as “use of assistants” and “flow of operation and forward planning” – which were assigned to the “collaborator” role within CanMEDS, and “situation awareness”, “communication and teamwork”, and “decision making” components within NOTSS. However, none of these studies reported on the non-technical skills item in their outcomes [30,31,34,37,41,42,46,47,50,52,53,56,58,60,61]. The NOTSS component ‘Leadership’ and the CanMEDS roles ‘Leader’, ‘Communicator’, ‘Health Advocate’ and ‘Professional’ were not reported or measured by any study.

### Methodological quality of included studies

There were only two studies with an overall low risk of bias (**Supplemental fig. 8**) [39,57]. All other studies had at least some concerns as they suffer from the lack of a pre-specified study protocol (*n* = 30), insufficient specification of the randomization process and/or insufficiently blinded outcome assessors (*n* = 27).

## Discussion

Research, development, and implementation of digital training tools for surgical residents has increased substantially in recent years, and has gained much attention during the COVID-19 pandemic. This systematic review and meta-analysis reveals that digital tools are widely and readily available, that most evidence is available for VR trainers, and that very few studies address non-technical skills. Most digital tools had positive effects on skill scores and performance time when compared to a control group, and significant effects of training factors were not observed in this study. While this study presents the best available evidence, caution is needed in interpreting these results due to high associated (>70 %) heterogeneity.

In this light, there are two results which can be interpreted with more certainty; VR trainers were equally effective as using a box trainer and as training in a wet or dry lab in this review. While the first outcome is accordance with earlier systematic reviews, no precedent of the latter is available in current literature [13]. Based on these results, box trainers, VR trainers and wet/dry labs are all valid training methods, yet there are differences to consider; wet/dry labs perform better with regard to training efficiency (the speed in which new skills are acquired), but do not have the advantage of training in your own time that the two digital tools have [34,55]. Box trainers are widely available in different configurations and from different manufacturers, are probably the least costly training tool of the three, yet are often primarily aimed at novices [52,63]. When the aim is to support residents in working more autonomously, clinically relevant training tools (such as wet/dry labs or VR trainers) may be necessary before the skills can be transferred to the OR [64]. VR-training does not have these disadvantages, but can be expensive and time consuming to develop [52]. Therefore, it is worth it to consider if there appropriate VR-systems are available, before deciding to develop a new system for a training objective.

Most studies in this review compared a digital tool with a control-group (receiving no additional training). While comparing an intervention with a placebo is a common and useful methodology in studies that evaluate medical interventions, this approach introduces several problems when it is used in educational research. Many digital tools had to be used in a structured way, dedicated time was provided, and the effects of their use was evaluated, while the control group received no additional training and none of this attention. While we believe embedding digital tools is of the utmost important to optimize their use, this difference in the provision of the intervention in this approach is problematic for the validity of the results. In essence, what all of these studies prove is that if resident training is monitored, skills will likely improve. Due to inherently introduced attention bias, it is unclear whether this effect originates from the digital tool itself or from the imposed training. A remarkable example of this is the study of Adams et al., who observed that it is more effective for technical skills acquisition to train on a gaming console than on a box trainer, provided that more hours are trained [65]. In subgroup analysis we therefore aimed to evaluate the effects of training structure and duration. While we found suggestions of differences in training effects of these factors, the effects were not significant and associated heterogeneity was high. While this makes it challenging to interpret outcomes, it clearly reveals the need to improve the quality of research on digital tools. “Proving” that a digital tool works in a study with these biases and unclarity should not be enough support to implement and adopt the tool in surgical curricula – let alone to use it as a way to improve training and its' efficiency.

We therefore highly advocate improving the robustness of studies on digital tools. A start would be to adhere to reporting guidelines (most studies suffered from overall risk of bias due to the lack of a protocol and information on randomization), and diminishing the effects of attention bias by providing equal training schedules to all interventions. Exemplary are the immersive VR studies which all compared the intervention with the reading of textbooks and journals [42,50,51,61]. When comparing these studies with the study by Orzech et al. [52] – who compared a box trainer with a VR trainer and with training in the OR, including a cost-analysis – the external validity and meaningfulness of the results of the latter are evident.

In recent years, it has become clear that a surgeon lacking non-technical skills, affects not only the performance in surgical teams, but may lead to avoidable incidents, and thus impact postoperative outcome [[15], [16], [17], [18],^66^]. However, there is little focus on teaching and evaluating non-technical skills [67]. No digital tools could be identified in this review, yet it seems improbable that these non-technical skills are not trained at all. Attitudes and non-technical skills are more likely to be trained on the job itself, or using non-digital simulation [68,69]. Yet there is no reason other than the blind-spot of the developer or educator not to develop tools to support both technical and non-technical skill, or not to evaluate the effect of digital resources on non-technical skills with the same objective methodology as their technical equivalent [67,70,71]. Promising technologies to this regard are VR, AR (augmented reality), MR (mixed reality) and telementoring solutions; as well as use of the Metaverse and medical data recorders in the OR. VR, AR and MR training have shown to increase both knowledge and motivation, and to provide insight in work ethics, personality, and communication skills of various trainees in medicine [72,73]. Additionally, telementoring can support both mentee and mentor, and reduce the strain of giving written feedback. Use of data output coming from a medical data recorder may help to qualify and, upon analysis, improve non-technical skills performance of surgical teams. It is known that using such a system benefits surgical teams and influences human factors that relate positively to performance of surgery [17,74].

Increasing scientific data related to the question if, and how, digital tools can help enhance the skills and traits as described in the intrinsic CanMEDS roles and NOTSS would be a first step [67]. Upcoming innovative educational tools such as virtual rounds, video-based learning, livestreamed surgical cases, Artificial Intelligence-based analysis of surgical performances, and many others tools and resources may prove invaluable in surgical resident training in the future [[75], [76], [77], [78]]. It is therefore up to surgical educators and residents to stay on top of these innovations and identify training requirements, thereby targeting specific didactic needs and providing a tailored education.

A possible approach to support digital training of non-technical skills in surgery is to follow the introduction template of the OSATS-checklist back in 1996 [79,80]. Projection of the OSATS approach onto the CanMEDS roles and NOTSS skills requires explication into standardized, measurable non-technical skill indicators – specific to surgical practice. An initial step would be to implement non-technical scoring systems digitally into surgical curricula. The result of combining this non-technical skills checklist with technical skills assessments such as the OSATS, will be a more comprehensive overall surgical skills assessment of the resident. Currently, new systems are being developed to digitally advance education and evaluation of technical and non-technical skills, such as use of the OR Black Box™ outcome report in which the rating scales are embedded, and immersive VR and MR training systems [74,81,82].

There are several limitations to this study. Included studies suffered from variation in study methodology, overall risk of bias and heterogeneity, and most studies suffered from confounding of novelty, availability, attention, and/or compliance – to name a few. While the meta-analyses are therefore of suboptimal value, we chose to perform them nevertheless to provide the best available evidence and reveal its limitations. Including studies which report on subjective outcomes may have resulted in identifying and including more studies focussing on non-technical skills. However, resources are consistently evaluated objectively on their technical outcomes in controlled studies. For them to be truly advantageous they need to be able to improve real life skills – including non-technical skills. We therefore believe that their effect on non-technical skills needs to be evaluated in the with the same methodological setup. Lastly, very little information is available on the effects of PGY on outcomes, only one study differentiated between different PGY's. They found inconsistent results, and their study was not powered on this outcome [41].

While the efficacy of digital tools in enhancing technical surgical skills is evident - especially for VR-trainers -, there is a lack of evidence regarding non-technical skills, and need to improve methodological robustness of research on new (digital) tools before they are implemented in curricula.

## CRediT authorship contribution statement

Conceptualisation: TF, MS; Data collection and analysis: TF, SvdS; Writing: TF; Supervision: JB, ENvD, MS; Editing: TF, SvdS, EB, JB, ENvD, MS.

## Ethical statement

Not applicable.

## Funding

This research did not receive any specific grant from funding agencies in the public, commercial, or not-for-profit sectors.

## Declaration of competing interest

The authors declare no conflict of interest.

## Appendix A Supplementary data

Supplementary figures Image 1
