# Supplementary material for: Which, how, and what? Using digital tools to train surgical skills; a systematic review and meta-analysis
Source: Surg Open Sci. 2023 Oct 4;16:100–10. doi: 10.1016/j.sopen.2023.10.002 (PMC10565595; doi:10.1016/j.sopen.2023.10.002)
Supplement: Supplementary file 1 — Supplementary figures [file mmc1.docx]

# Supplemental material

#
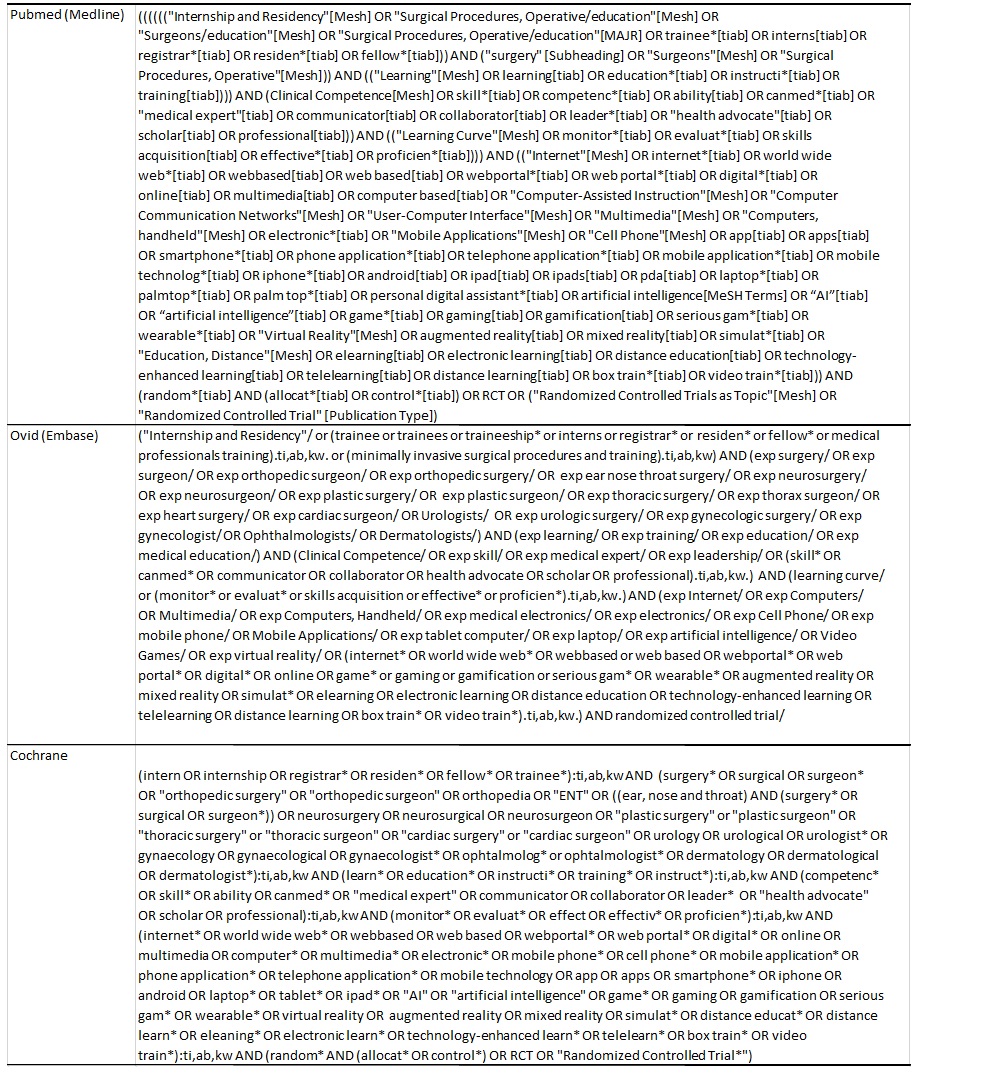


Supplemental figure 1: Search strings for PubMed (Medline), Ovid (Embase), and cochrane engines


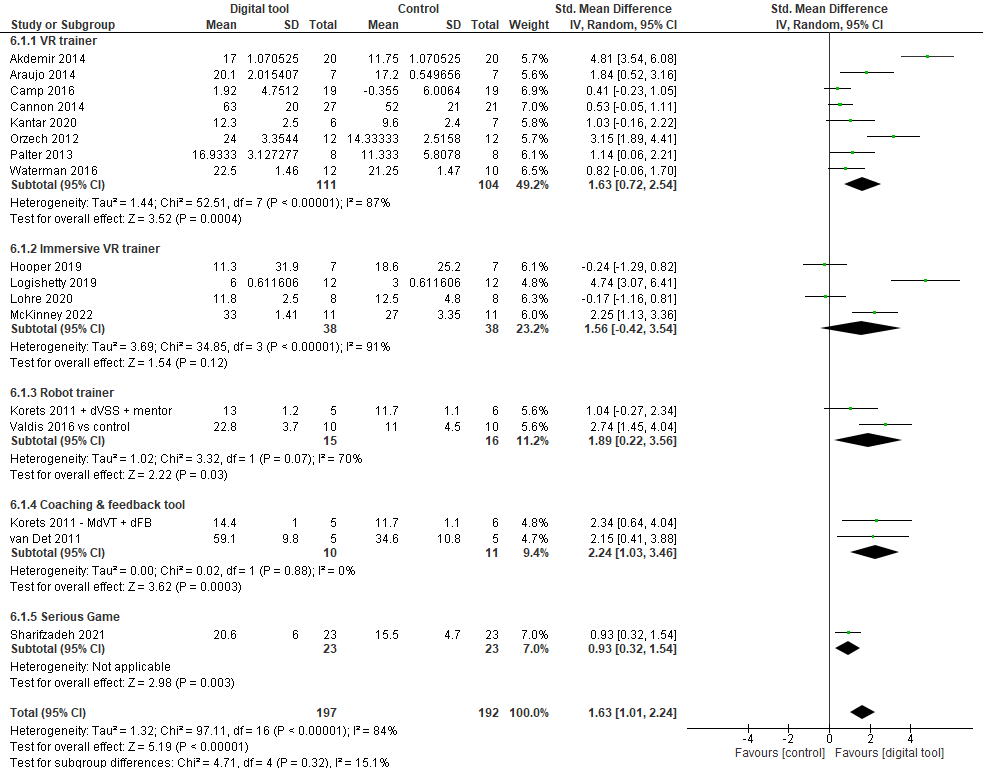


Supplemental figure 2: Subgroup analysis of digital tools versus control group based on skill scores


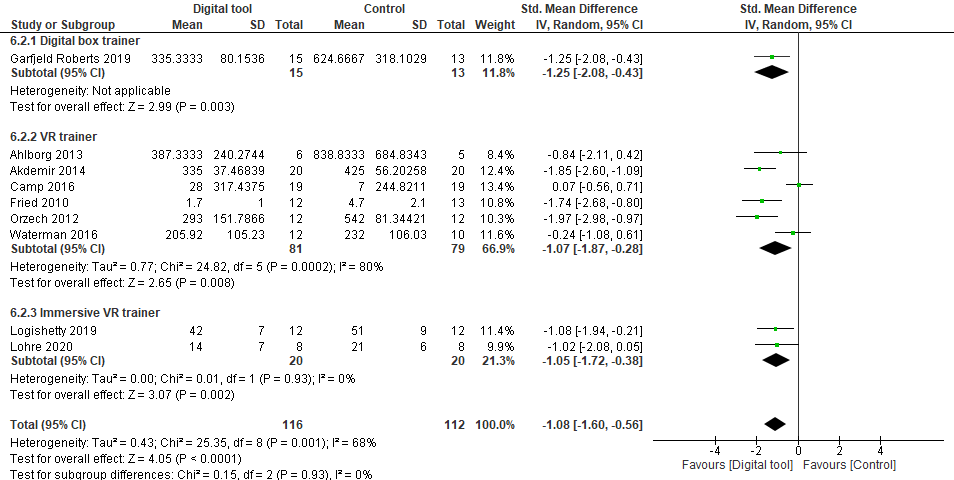


Supplemental figure 3: Subgroup analysis of digital tools versus control group based on time outcomes


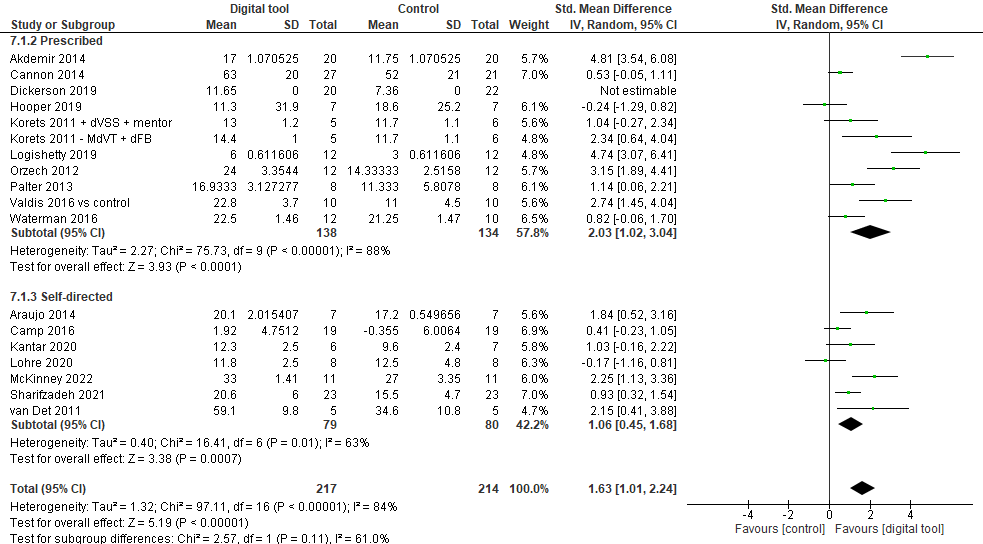


Supplemental figure 4: Subgroup analysis of training structure of digital tools vs control group on skill outcomes


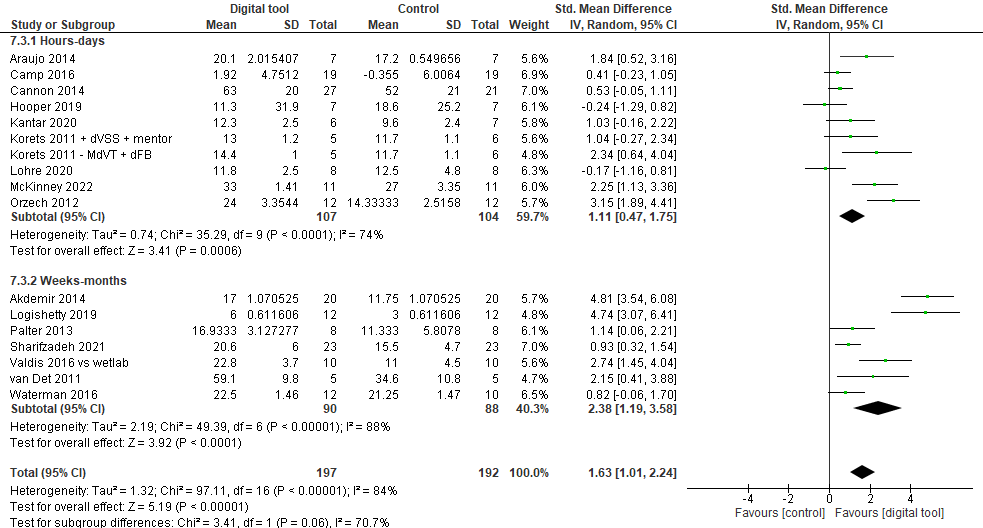


Supplemental figure 5: Subgroup analysis of training duration of digital tools vs control group on skill outcomes


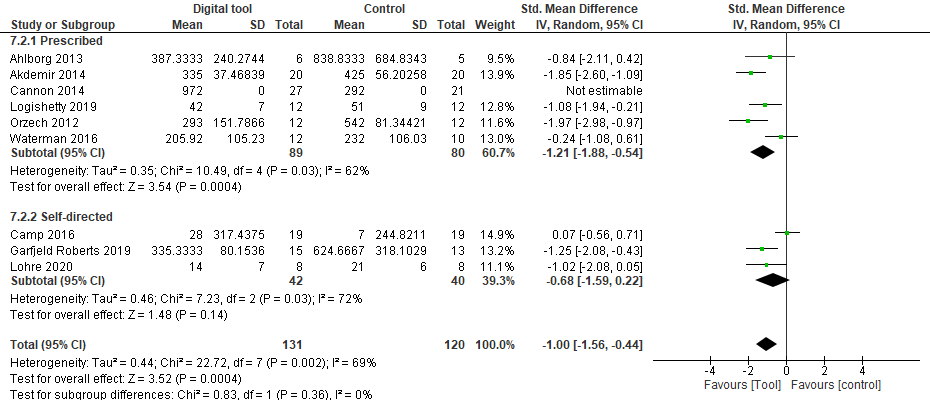


Supplemental figure 6: Subgroup analysis of training structure of digital tools vs control group on performance time


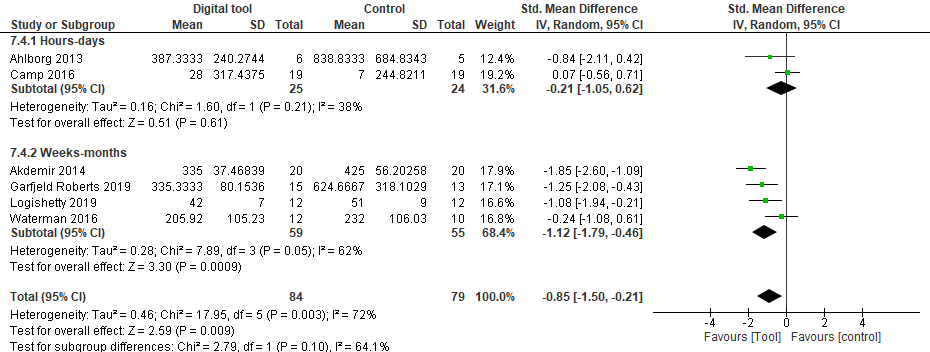


Supplemental figure 7: Subgroup analysis of training duration of digital tools vs control group on performance time

Supplemental figure 8: Risk of Bias of included studies according to the Revised Cochrane risk-of-bias tool for randomized trials (RoB 2)
